# Neurologically Symptomatic Pneumorrhachis as the Primary Clinical Manifestation of Rectal Cancer

**DOI:** 10.1007/s00062-025-01530-7

**Published:** 2025-05-27

**Authors:** Sabine Sari, Natascha Wallendszus, Tobias Struffert

**Affiliations:** 1 Institute of Neuroradiology, Justus Liebig-University Gießen, Klinikstraße 33, 35392 Gießen, Germany; 2 Institute of Neurosurgery, Justus Liebig-University Gießen, Klinikstraße 33, 35392 Gießen, Germany

## Introduction

Pneumorrhachis (PR) is defined as the presence of air in the spinal canal and was initially described by Gordon et al. in 1977 [1]. The etiology of PR is categorized into iatrogenic, non-traumatic, or traumatic [2]. PR can be anatomically classified into intradural (subdural or subarachnoid) or extradural (epidural) PR and is associated with different pathophysiologic mechanisms and causes. It has a relevant medical implication whether the air in PR is present in the intradural or extradural space. Air in the epidural space is mainly seen after traumatic injury, nontraumatic injury to the respiratory or gastrointestinal system, or is induced iatrogenically. Epidural PR is usually considered benign since it is primarily asymptomatic and resolves spontaneously [3]. Subarachnoid PR, on the other hand, is a severe condition that is usually accompanied by neurological symptoms and even mortality. Subarachnoid PR is nearly always associated with a pneumocephalus, has a high risk of infection, and may induce changes in the intracranial and intraspinal pressure since the air in the subarachnoid space can move up and down [4, 5].

## Clinical Case

**Fig. 1:**
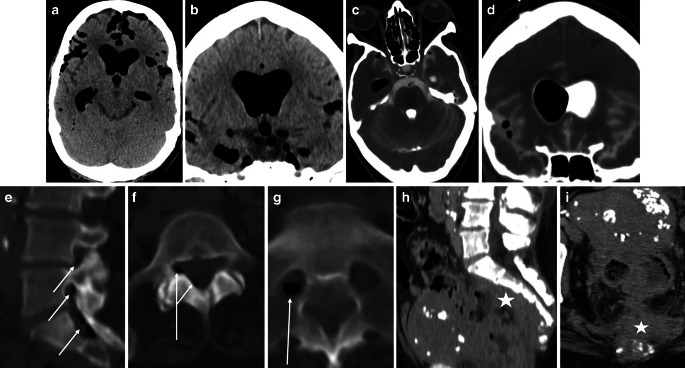
**a**, **b** Plain cranial CT scans of the ubiquitous air in the subarachnoid space and the ventricular system: **a** axial view, **b** coronal view. **c**, **d** Contrast-enhanced cisternography: **c** axial view, **d** coronal view. **e**–**g** Plain CT scan of the intradural lumbosacral pneumorrhachis (*white arrows*): **e** sagittal view, **f** axial view L5, **g** axial view S1. **h**, **i** Contrast-enhanced abdominal CT of the tumor and abscess in the retroperitoneal cavity and anterior sacral surface (*white star*): **h** sagittal view, **i** axial view

We here describe a case of a 73-year-old female with no history of recent trauma, surgery, or lumbar puncture. She had been sensing lumbar pain recently, which she had ascribed to her gardening. Due to a collapse, the patient was admitted to the emergency department with clinical symptoms of confusion, aphasia, and motoric restlessness. Body temperature on admission was 38.4 °C. Blood test on admission revealed an elevated inflammatory response (WBC 20.9 giga/l, CRP 119.92 mg/l). Suspecting meningitis, a cranial CT scan was initiated, and a systemic antibiotic treatment was started with Meropenem and Vancomycin. The CT scan of the head revealed a massive amount of ubiquitous air in the subarachnoid space and the ventricular system without any evidence of bony destruction or fracture of the skull or skull base (Fig. 1a, b). Cranial CT follow-up showed a progressive dilatation of the ventricles. Therefore, an intraventricular drain was implanted for pressure relief. CSF samples were collected, which never revealed any proof of intrathecal bacteria but showed inflammatory changes in the CSF (cell count 386, protein 1010 mg/l). Meanwhile, Staphylococcus epidermidis was detected in blood samples. Via the intraventricular drain, a cisternography was performed to rule out a cranial dural tear, but it was negative (Fig. 1c, d). In addition, a thoracic and abdominal CT scan depicting the entire dural tube was performed to rule out an injury to the respiratory or gastrointestinal system. An intraspinal pneumorrhachis was detected on the lumbar and sacral levels. Along the right nerve root S1, a collection of air was depicted, connecting to the spinal canal. Moreover, the CT scan revealed a presacral mass lesion with bony sacral destruction and air pockets (Fig. 1e–i). Abdominal surgery was done, revealing a tumor originating from the rectosigmoid and infiltrating the vagina as well as the sacrum associated with an abscess. Due to its extensive infiltration, it was not possible to remove the tumor in toto, so a resection of the rectum and the sigmoid colon with implementation of a descendostoma and closure of the perineum was performed. Histopathological findings revealed an adenocarcinoma of the rectum in the state pT4a, N1 (2/14), L0, V0, Pn1, and a resection status of the category R2. The management with antibiotics was changed to Ciprofloxacin and Metronidazole. The patient had a resolution of fever and the initial clinical symptoms, so she could be discharged on the 19th day. Follow-up CT scans of the head revealed complete resorption of the ubiquitous air in the subarachnoid space and the ventricular system. Further, the patient regularly attended the oncological outpatient clinic for clinical evaluations. She reported well-being, and CT scans of the whole body showed stable disease. An interdisciplinary tumor board recommended a combined radio-chemotherapy.

## Discussion

According to the current guideline, a computerized tomography (CT) scan of the head was performed since the patient initially presented with confusion, aphasia, and elevated inflammatory markers [6]. The CT scan of the head revealed a massive amount of ubiquitous air in the subarachnoid space and the ventricular system. This condition was first described in 1741 by Lecat and coined “pneumocephalus” in 1913 by Luckett [7]. Head trauma is up to 74% of the cause of pneumocephalus since air can enter the cranial cavity through skull fractures [8]. However, if a bony destruction or fracture of the skull or skull base, as well as a dural tear, could be ruled out, further investigations are necessary, such as a thoracic and abdominal CT scan depicting the whole dural tube to rule out a nontraumatic injury of the respiratory or gastrointestinal system as the cause of the pneumocephalus. In the presented case, the abdominal CT scan showed a presacral mass lesion with bony sacral destruction and air pockets, revealing the origin of the pneumorrhachis. The air from the rectum had found its way into the retroperitoneal space and via the sacral foramen into the spinal canal, causing a sacral and lumbar pneumorrhachis. Apart from our case, there have been two additional reported cases of pneumorrhachis associated with cancer of the colon or rectum [9, 10].

The air rose in the spinal canal and only secondarily caused a pneumocephalus. This circumstance also explained the inflammatory changes of the CSF, causing secondary meningitis with respective neurologic symptoms. This supports the indication for a broad antibiotic therapy when there is an assumption of a pneumocephalus or pneumorrhachis. As far as possible, the underlying cause of the pneumorrhachis should be resolved, which was done in our case by subtotal resection of the presacral mass lesion. To rule out secondary complications such as hydrocephalus, cranial CT follow-ups are essential, which fortunately showed in our patient complete resorption of the intracranial air. Furthermore, thoracic and abdominal CT scan follow-ups are necessary to ensure the PR’s underlying cause has been fixed.

## Conclusion

This case impressively shows the necessity of a thorough search for a dural impairment in the case of spontaneous pneumocephalus. Although in 74% of all cases, the reason is a skull base fracture with an associated dural tear, one has to consider the further depiction of the entire dural tube if the cranial imaging does not reveal a suitable pathology. CT remains the gold standard for detecting fractures, depicting PR, and assessing the adjacent anatomy in the search for a non-traumatic cause. Furthermore, the obligation of immediate broad antibiotic therapy in the case of a pneumocephalus is emphasized since the impaired dural sheet leads to a connection between the sterile intradural compartment and the unsterile surrounding, which is a source of infection. Finally, secondary complications must be considered, and the PR’s primary cause must be fixed in the long term.

## Data Availability

The data of this study are available from the corresponding author on reasonable request.
